# An In Vitro and In Silico Study of the Enhanced Antiproliferative and Pro-Oxidant Potential of *Olea europaea* L. cv. Arbosana Leaf Extract via Elastic Nanovesicles (Spanlastics)

**DOI:** 10.3390/antiox10121860

**Published:** 2021-11-23

**Authors:** Taghreed S. Alnusaire, Ahmed M. Sayed, Abeer H. Elmaidomy, Mohammad M. Al-Sanea, Sarah Albogami, Mha Albqmi, Bassam F. Alowaiesh, Ehab M. Mostafa, Arafa Musa, Khayrya A. Youssif, Hesham Refaat, Eman M. Othman, Thomas Dandekar, Eman Alaaeldin, Mohammed M. Ghoneim, Usama Ramadan Abdelmohsen

**Affiliations:** 1 Biology Department, College of Science, Jouf University, Sakaka 72341, Saudi Arabia; tasalnosairi@ju.edu.sa (T.S.A.); bfalawish@ju.edu.sa (B.F.A.); 2 Department of Pharmacognosy, Faculty of Pharmacy, Nahda University, Beni-Suef 62513, Egypt; ahmed.mohamed.sayed@nub.edu.eg; 3 Department of Pharmacognosy, Faculty of Pharmacy, Beni-Suef University, Beni-Suef 62511, Egypt; Abeer011150@pharm.bsu.edu.eg; 4 Pharmaceutical Chemistry Department, College of Pharmacy, Jouf University, Sakaka 72341, Saudi Arabia; mmalsanea@ju.edu.sa; 5 Department of Biotechnology, College of Science, Taif University, P.O. Box 11099, Taif 21944, Saudi Arabia; dr.sarah@tu.edu.sa; 6 Chemistry Department, College of Science and Arts, Jouf University, P.O. Box 756 Alqurayyat, Saudi Arabia; maalbgmi@ju.edu.sa; 7 Pharmacognosy Department, College of Pharmacy, Jouf University, Sakaka 72341, Saudi Arabia; emmoustafa@ju.edu.sa; 8 Department of Pharmacognosy, Faculty of Pharmacy, Al-Azhar University, Cairo 11371, Egypt; 9 Department of Pharmacognosy, Faculty of Pharmacy, Modern University for Technology and Information, Cairo 11865, Egypt; khayrya.youssif@pharm.mti.edu.eg; 10 Department of Pharmaceutics, Faculty of Pharmacy, Deraya University, Minia 61111, Egypt; hesham.refaat@deraya.edi.eg (H.R.); eman.alaa@deraya.edu.eg (E.A.); 11 Department of Biochemistry, Faculty of Pharmacy, Minia University, 61519 Minia, Egypt; eman@toxi.uni-wuerzburg.de; 12 Department of Bioinformatics, Biocenter, University of Wuerzburg, Am Hubland, 97074 Wuerzburg, Germany; dandekar@biozentrum.uni-wuerzburg.de; 13 Department of Pharmaceutics, Faculty of Pharmacy, Minia University, Minia 61519, Egypt; 14 Department of Pharmacy Practice, College of Pharmacy, Al Maarefa University, Ad Diriyah 13713, Saudi Arabia; mghoneim@mcst.edu.sa; 15 Department of Pharmacognosy, Faculty of Pharmacy, Deraya University, New Minia 61111, Egypt; 16 Department of Pharmacognosy, Faculty of Pharmacy, Minia University, Minia 61519, Egypt

**Keywords:** olive, *Olea*, metabolomic profiling, antiproliferative, pro-oxidant, encapsulation, spanlastic, nanocarrier, docking, molecular dynamics simulation

## Abstract

The olive tree is a venerable Mediterranean plant and often used in traditional medicine. The main aim of the present study was to evaluate the effect of *Olea europaea* L. cv. Arbosana leaf extract (OLE) and its encapsulation within a spanlastic dosage form on the improvement of its pro-oxidant and antiproliferative activity against HepG-2, MCF-7, and Caco-2 human cancer cell lines. The LC-HRESIMS-assisted metabolomic profile of OLE putatively annotated 20 major metabolites and showed considerable in vitro antiproliferative activity against HepG-2, MCF-7, and Caco-2 cell lines with IC_50_ values of 9.2 ± 0.8, 7.1 ± 0.9, and 6.5 ± 0.7 µg/mL, respectively. The encapsulation of OLE within a (spanlastic) nanocarrier system, using a spraying method and Span 40 and Tween 80 (4:1 molar ratio), was successfully carried out (size 41 ± 2.4 nm, zeta potential 13.6 ± 2.5, and EE 61.43 ± 2.03%). OLE showed enhanced thermal stability, and an improved in vitro antiproliferative effect against HepG-2, MCF-7, and Caco-2 (IC_50_ 3.6 ± 0.2, 2.3 ± 0.1, and 1.8 ± 0.1 µg/mL, respectively) in comparison to the unprocessed extract. Both preparations were found to exhibit pro-oxidant potential inside the cancer cells, through the potential inhibitory activity of OLE against glutathione reductase and superoxide dismutase (IC_50_ 1.18 ± 0.12 and 2.33 ± 0.19 µg/mL, respectively). These inhibitory activities were proposed via a comprehensive in silico study to be linked to the presence of certain compounds in OLE. Consequently, we assume that formulating such a herbal extract within a suitable nanocarrier would be a promising improvement of its therapeutic potential.

## 1. Introduction

*Olea europaea* is a venerable cultural plant habitat in the Mediterranean [1]. The cultivation of this tree extends back many thousands of years and perhaps took place in the Eastern Mediterranean [1]. More than 300 original olive varieties have been developed worldwide to produce olive products [1]. These outputs represent a significant branch of industry in the Mediterranean regions, especially Spain, Italy, and Greece [1]. *O. europaea* is a critical tree, which requires limited demands on the soil and atmosphere and is several hundred years old [1].

Olive fruits and leaves are well-known for their therapeutic potential in many diseases [2]. The therapeutic utilities of olive trees, particularly their leaves, have been pointed out in folk medicine [3]. Studies have shown that it can reduce blood glucose level, serum cholesterol, and uric acid [3]. Additionally, olive fruits treat diabetes, elevated blood pressure, inflammatory disorders, diarrhea, respiratory tract disorders, urinary tract infections, intestinal diseases, and hemorrhoids [1,2,3,4]. Many phenolic olive leaf-derived compounds, principally iridoids and secoiridoids [4], and their pharmacological potentiality have been the focus of concern for investigation in the last decade [5,6]. Olive fruits are edible [7], with many biologically phenolic compounds [8]. Volatile compounds from *O. europaea* fruits and leaves and their functions in aroma improvement have also been an intense field of modern research [9].

Despite the vast range of therapeutic effects of such components, their optimum use has been hindered by low stability under different pH and temperature conditions, their hydrophobicity, and compliance due to the bitter taste [10]. Therefore, there was an essential need to design advanced delivery systems to cope with these aspects of these components. Many nanocarriers were developed to promote the bioavailability and therapeutic potential of naturally active products and their components [11,12,13]. The liposome of *O. europaea* extract was recently formulated by González-Ortega et al., 2020 [14], who reported enhanced stability for the extract components when encapsulated within a lipid-based formulation.

Spanlastics are nanovesicular systems that are formulated using Spans with an edge activator. Many active agents can be enclosed in the bilayer formed by the nonionic surfactants [15]. Spanlastics show comparable stability compared to other forms such as liposomes. Unlike other nanocarriers that contain cationic surfactants, they are nonirritant and provide improved delivery due to their deformable elastic nature [16].

During the last ten years, many reports have outlined the positive correlation between the pro-oxidant capacity and the antiproliferative effects of several chemical substances, particularly herbal extracts and polyphenolic compounds [17,18,19,20,21].

Across the world, cancer has a major impact on society. Cancer reports clearly show the aggressiveness of cancer and the difficulty of investigation in the initial stages. Prevention through new strategies is a challenge. Currently, the recognition of modern remedial strategies concentrating on inhibiting tumor cell proliferation has grown into a significant demand. In recent decades, the research of the anticancer properties of food ingredients has received further attention. Recently, researchers have been focusing on engaging in the benefits of the Mediterranean diet, longevity, and quality of life [21].

Herein, we aimed to evaluate the effect of OLE and its encapsulation within the spanlastic nanoformulation on the improvement of its pro-oxidant and, in turn, its antiproliferative activity toward HepG-2, MCF-7, and Caco-2 cancer cell lines. Additionally, we integrated LC-HRMS metabolic profiling along with several in silico studies (e.g., inverse docking, molecular dynamic simulation, binding free energy calculations) to highlight the main bioactive components and suggest a probable mode of action.

## 2. Materials and Methods

### 2.1. Plant Material

*O. europaea* leaves were collected in April 2020 from Basita farms, Aljouf, KSA. It was identified by Hamdan Ogereef, Camel and Range Research Center, Sakaka, Saudi Arabia. The samples are kept under voucher specimen number (2020-BuPD 75) at Pharmacognosy Department, Faculty of Pharmacy, Beni-Suef University, Egypt. According to the departmental/institutional/local/national guidelines in Egypt, permissions and approvals are not required on plant research.

### 2.2. Chemicals and Reagents

Chemicals consumed in this study, if not mentioned, were purchased from Sigma-Aldrich (St. Louis, MO, USA). Doxorubicin, the anticancer-positive control, was supplied from Merck Company, Germany. WI-38, HepG-2, MCF-7, and Caco-2 cancer cell lines were from the American Type Culture Collection and continuously subcultured twice per week. WI-32 (normal human fibroblasts) and HepG-2 (human liver cancer) cell lines were cultured in Eagle’s Minimum Essential medium (EMEM) at 37 °C, supplemented with 10% (*v/v*) fetal bovine serum (FBS), 1% (*w/v*) L-glutamine, and 0.4% (*w/v*) antibiotics (50 IU/mL of penicillin, 50 IU/mL of streptomycin).

The MCF-7 (human breast adenocarcinoma) cell line was cultured at 37 °C and 5% (*v/v*) CO_2_ in RPMI 1640 medium, and it was supplemented with 5% (*v/v*) fetal bovine serum (FBS), 1% (*w/v*) L-glutamine, 1% (*v/v*) sodium pyruvate, and 0.4% (*w/v*) antibiotics (50 IU/mL of penicillin, 50 IU/mL of streptomycin).

The Caco-2 (human colon adenocarcinoma) cell line was cultured at 37 °C and 5% (*v/v*) CO_2_ in modified Eagle’s medium (MEM) with modified Earle’s salts, and it was supplemented with 20% (*v/v*) fetal bovine serum (FBS), 1% (*w/v*) L-glutamine, 1% (*v/v*) sodium pyruvate, 1% (*v/v*) nonessential amino acids, and 0.4% (*w/v*) antibiotics (50 IU/mL of penicillin, 50 IU/mL of streptomycin).

### 2.3. Extraction of Plant Material

*O. europaea* leaves (2 kg) were collected, thoroughly washed, and air-dried in shade for 10 days. They were subjected to grinding using an OC-60B/60B herbal grinding machine (60–120 mesh, Henan, Mainland China). The ground plants were macerated for exhaustive extraction with 70% EtOH (5L X3) at room temperature, evaporated, and concentrated in vacuo at 45 °C using a rotary evaporator (Buchi Rotary evaporator R-300, Cole-Parmer, USA) to obtain 80 g of dry extract, which was stored at 4 °C for further biochemical studies. Our investigations of the water-based extracts are underway in order to obtain such a bioactive extract using a greener solvent.

### 2.4. Metabolic Profiling

*O. europaea* leaf crude ethanolic extract was developed, and approximately 1 mg/mL of the extract was used for LC-mass investigation. The concentrated ethanolic extract was exposed to metabolic analysis using LC-HRMS, in correspondence with our previous report [22], on an Acquity Ultra Performance Liquid Chromatography system coupled to a Synapt G2 HDMS quadrupole time-of-flight hybrid mass spectrometer (Waters, Milford, MA, USA). An Acquity ultra-performance liquid chromatography system attached to a Synapt G2 HDMS quadrupole time-of-flight hybrid mass spectrometer (Waters, Milford, MA, USA) was employed. Positive and negative ESI ionization modes were employed to obtain the high-resolution mass spectrometry connected with a spray voltage of 4.5 kV, capillary heat of 320 °C, and mass charge ratios of 150–1500 m/z. The MS dataset was processed, and data were obtained using MZmine 2.20 based on the approved parameters. Mass ion peaks were identified and followed by chromatogram builder and chromatogram deconvolution. The local minimum search algorithm was utilized, and isotopes were further determined via the isotopic peaks’ grouper. Missing peaks were illustrated using the gap-filling peak finder. An adduct investigation along with a complex search was performed. The refined dataset was later exposed to molecular formula prediction and peak identification. The positive and negative ionization mode datasets from the respective extract were dereplicated against the DNP (Dictionary of Natural Products) databases.

### 2.5. Preparation of OLE-Containing Spanlastic

Spanlastics were prepared with the spraying technique reported previously by Refaat et al., 2019 [12]. Briefly, the organic phase was prepared by dissolving Span 60, Tween 80 (4:1 molar ratio), and the alcoholic extract of *O. europaea* (150 mg) in 2 mL of absolute ethanol. Sucrose was dissolved in 3 mL of deionized water to yield a 9% *w/v* sucrose solution (aqueous solution). The organic phase was sprayed using a modified long nozzle sprayer on the surface of the aqueous phase (100 µL for 5 s). During spraying, the aqueous phase temperature was kept constant at 60 °C with stirring at 1500 rpm. Stirring at 60 °C was maintained for 20 min until spanlastics were spontaneously formed and ethanol was completely evaporated. The obtained spanlastics were stored at 4 °C for 24 h to guarantee the complete annealing of the formed bilayer. Those results are consistent with a previous report that revealed that olive extract is appropriate only for the low-temperature thermal application for food and cosmetics because of its low thermal stability.

### 2.6. Characterization of the Prepared Spanlastics

#### 2.6.1. Determination of Vesicle Size, Size Distribution, and Zeta Potential

The OLE-containing spanlastics’ size and polydispersity index were determined with Zetasizer Nano ZSP (Malvern Instruments, Malvern, UK). Spanlastics were analyzed at 25 °C after dilution with deionized water. Procedures were repeated in a triplicate manner and average values were determined [23]. The zeta potential of the Millipore water-diluted spanlastics was evaluated using a Mastersizer (3000 E Malvern Instruments, UK). The average zeta potential was determined [24].

#### 2.6.2. Transmission Electron Microscopy Analysis (TEM)

The morphology and shape of the prepared spanlastics were investigated using electron microscopy (JEM-1400, Jeol, Tokyo, Japan) maintained at 80 kV. For this, a spanlastic suspension was placed onto a carbon-coated copper grid. The carbon-coated grid was kept for 10 min at ambient temperature before examination [12,25].

#### 2.6.3. Entrapment Efficacy (%EE) of the Prepared Liposomes

The entrapped % of the extract, calculated as % of flavonoid entrapment, within the formulation was determined according to Refaat et al., 2019 [12]: washing of the formulation was performed by the centrifugation of 1 mL of spanlastic suspension at 4 °C, 15,000 rpm for 1 h. The separated spanlastics were twice rewashed by dilution with distilled water followed by centrifugation until the complete removal of the unentrapped extract. The prepared spanlastics were destructed by sonication with absolute ethanol and then centrifugation at 15,000 rpm for 30 min. The percentage (%) of trapped flavonoids was determined as described by Refaat et al., 2019 [12]. Briefly, equal volumes of 10% aluminum chloride alcoholic solution and the supernatant were mixed and diluted with absolute alcohol with a final volume of 2 mL. The absorbance was determined against blank at λmax 410 nm using a UV/Vis spectrophotometer (Spectronic Genesys^®^), with Winspec Software. The mean % of entrapped flavonoids was determined using the equation:

$$\%\mathrm{EE}=\frac{\left(amount\;of\;entrapped\;flavonoids\;\right)}{\left(total\;amount\;of\;loaded\;flavonoids\;\right)\;}\text{×}100$$

#### 2.6.4. Characterization of the Heat Effect

The relative change in the weight of olive extract and prepared spanlastics relative to the temperature change was studied using Thermogravimetric Analysis (TGA). Twenty milligrams of the dried samples were placed in a platinum pan and heated from 30 to 450 °C with a heat flow rate of 20 °C/min and nitrogen flow rate of 20 mL/min. Fourier-transform infrared (FT-IR) measurements over the wavenumber range from 4000 to 400 cm (Nicollet IS 10 FTIR spectrometer, USA) for the olive extract, blank spanlastics, and olive spanlastics were carried out after dispersion of the samples in KBr discs to study the possible interactions and bonding pattern between Span 60, Tween 80, and olive extract.

### 2.7. In Vitro Study

#### 2.7.1. In Vitro Antiproliferative Assay

The antiproliferative activity of OLE either free or encapsulated within the spanlastic dosage form was determined by the sulforhodamine B (SRB) assay as mentioned by Skehan et al. 1990 [26], and Vichai and Kirtikara 2006 [27], on the normal fibroblast (WI-32), breast (MCF-7), liver (HepG2), and colorectal (Caco2) cancer cell lines. Cells were cultivated in 96-well microtiter plates at an initial concentration of 1 × 10^5^ cells/well in 150 µL of fresh medium and left for 24 h to adhere to the wells. Concentrations of 0, 5, 12.5, 25, and 50 µg/mL of the respective extract were included. The plates were incubated for 48 h. The cells were fixed with 50 μL of cold trichloroacetic acid (10% final concentration) for 1 h at 4 °C. The plates were cleaned with distilled water (automatic washer Tecan, Germany) and stained with 50 μL of 0.4% SRB dissolved in 1% acetic acid for 30 min, at room temperature. Later, they were washed with 1% acetic acid and air-dried. The dye was solubilized with 100 μL/well of 10 M of tris base (pH 10.5). The optical density of each well was measured spectrophotometrically at 570 nm using an ELISA microplate reader (Sunrise Tecan reader, Germany). Doxorubicin was used as a positive control. The mean background absorbance was automatically subtracted, and mean values of each drug concentration were determined. The experiment was rerun three times, and later, the IC_50_ values were measured.

#### 2.7.2. Measurement of Intracellular Free Radicals

For the in vitro examination of the potential activity of OLE and OLE-spanlastics as pro-oxidants, 2′,7′-dichlorodihydrofluorescein diacetate (H_2_DCF-DA) dye was applied. Briefly, upon entry into live cells, acetate groups are removed from the H_2_DCF-DA by the action of the cellular esterases; then, oxidation of the dye to the fluorescent product dichlorofluorescein (DCF) in the presence of free radicals occurs and represents a marker for oxidative stress and pro-oxidant activity. The Caco-2 cells are used here as an example due to their high sensitivity to oxidative stress.

Generally, Caco-2 cells (8 × 10^5^ cells/µL) were treated with the test materials (7 µg/mL) for 1 and 10 min, and then treated with 20 µM of DCFDA for 30 min. After several washes in PBS, the DCF fluorescence was measured using flow cytometric analysis with excitation and emission wavelengths at 495 and 530 nm, respectively. All activity measurements were repeated three times for each test material concentration.

#### 2.7.3. Glutathione Reductase Enzyme Activity

The inhibitory activity of OLE and OLE-spanlastics against glutathione reductase (GR) was measured colorimetrically using the manufacturer protocol (abcam, Cat.No: ab8346). Briefly, the test material was dissolved in dimethyl sulfoxide to produce a stock solution of 1 mg/mL. The reaction mixture consisted of 100 mM of potassium phosphate buffer at pH 7.4, 1 mM of GSSG, 0.1 mM of NADPH, and 25 μL of GR. The test material was added from the stock solution by serial dilutions to give final inhibitor concentrations between 0.05 µg/mL and 50 µg/mL. GR activity was measured at 405 nm at 37 °C. All activity measurements were repeated three times for each test material concentration.

#### 2.7.4. Superoxide Dismutase Activity

The inhibitory activity of OLE and OLE-spanlastics against SOD was measured colorimetrically using the manufacturer’s protocol (abcam, Cat.No: ab65354). Briefly, the test material was dissolved in dimethyl sulfoxide to produce a stock solution of 1 mg/mL. The reaction mixture consisted of 100 mM of potassium phosphate buffer at pH 7.4, 1 mM of formazan dye, 25 μL of xanthine oxidase (to produce superoxide anions), and 25 μL of SOD solution. The test material was added from the stock solution by serial dilutions to give final inhibitor concentrations between 0.05 µg/mL and 50 µg/mL. SOD activity was measured at 450 nm at 37 °C. All activity analyses were repeated three times for each test material concentration.

### 2.8. In Silico Investigation

#### 2.8.1. Determination of the Potential Protein Targets

Potential protein targets for the OLE identified compounds were proposed by subjecting all these compounds to inverse docking against all proteins hosted in Protein Data Bank (PDB; https://www.rcsb.org/ accessed on 21 October 2021). idTarget platform (http://idtarget.rcas.sinica.edu.tw/ accessed on 21 October 2021) was used for this task. This structural-based screening software applies a unique docking approach called divide-and-conquer docking that adaptively builds small overlapping grids to make the search space on the protein surfaces more constrained, and hence, it can run a huge number of accurate docking experiments in a reduced time. The retrieved results were obtained as a list of binding affinity scores arranged from the highest negative value to the lowest one. We set a binding affinity score of −7 kcal/mol as a cut-off value to select the best targets for each identified compound in OLE. Considering the human proteins related to cancer pathogenesis, GR (PDB: 1BWC) and SOD (PDB: 5YTO) were selected for further modeling study.

#### 2.8.2. Molecular Dynamic Simulation and Binding Free Energy Calculation

The binding free energy calculation (Δ*G*) and molecular dynamic simulation were conducted as previously described in [28,29,30]. Desmond v. 2.2 software was used for performing MDS experiments. This software applies the OPLS force field. Protein systems were built using the System Builder option, where the protein structure was embedded in an orthorhombic box of TIP3P water together with 0.15 M of Na^+^ and Cl^−^ ions in 20 Å solvent buffer. Afterward, the prepared systems were energy-minimized and equilibrated for 10 ns. Desmond software automatically parameterizes inputted ligands during the system building step according to the OPLS force field. For simulations performed by NAMD, the parameters and topologies of the compounds were determined either using the Charmm27 force field with the online software Ligand Reader and Modeler (http://www.charmm-gui.org/?doc=input/ligandrm; accessed on: 29 October 2021) or using the VMD plugin Force Field Toolkit (ffTK). Afterward, the generated parameters and topology files were loaded to VMD to readily read the protein–ligand complexes without errors and then to conduct the simulation step.

#### 2.8.3. Binding Free Energy Calculations

Binding free energy calculations (∆*G*) were performed using the free energy perturbation (FEP) method. Briefly, this method calculates the binding free energy ∆*G*_binding_ according to the following equation: ∆G_binding_ = ∆G_Complex_ − ∆G_Ligand_. The value of each ∆*G* is estimated from a separate simulation using NAMD software. All input files required for simulation by NAMD can be papered by using the online website Charmm-GUI (https://charmm-gui.org/?doc=input/afes.abinding, accessed on 18 May 2021). Subsequently, we can use these files in NAMD to give the expected simulations using the FEP calculation function in NAMD. The equilibration was achieved in the NPT ensemble at 300 K and 1 atm (1.01325 bar) with Langevin piston pressure (for “Complex” and “Ligand”) in the presence of the TIP3P water model. Then, 10 ns of FEP simulations were performed for each compound, and the last 5 ns of the free energy values was measured for the final free energy values. Finally, the generated trajectories were visualized and analyzed using VMD software.

### 2.9. Statistical Analysis

All in vitro analyses were performed in triplicate. Pooled data were given as the mean ± standard error of the mean (SEM) of at least three separate experiments. The variations among diverse management groups were established by ANOVA, accompanied by Dunnett’s test using PASW Statistics^®^ version 18 (Quarry Bay, Hong Kong). A variation of *p* < 0.001 was considered statistically significant correlated with a vehicle-treated control group and illustrated by a * symbol. The IC_50_ values were established utilizing a nonlinear regression curve fitting analysis using GraphPad Prism software version 6 (La Jolla, CA, USA).

## 3. Results and Discussion

### 3.1. Dereplication of Olea europaea L. cv. Arbosana

**Figure 1:**
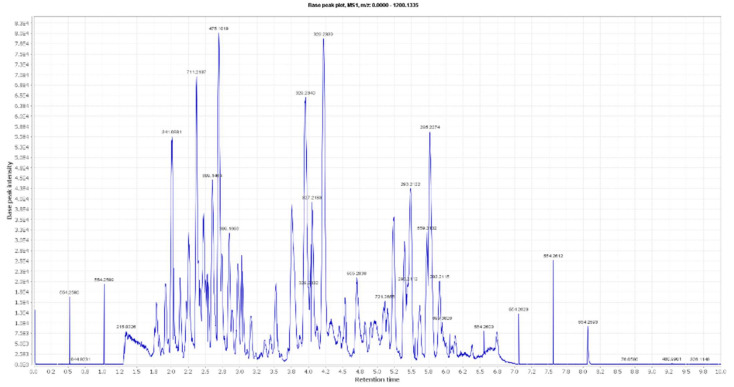
LC-HRESIMS chromatogram of the dereplicated metabolites of *Olea europaea* (Positive).

**Figure 2:**
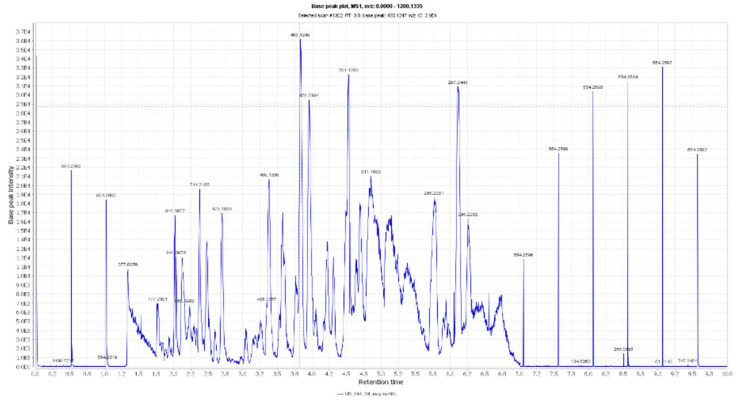
LC-HRESIMS chromatogram of the dereplicated metabolites of *Olea europaea* (Negative).

**Figure 3:**
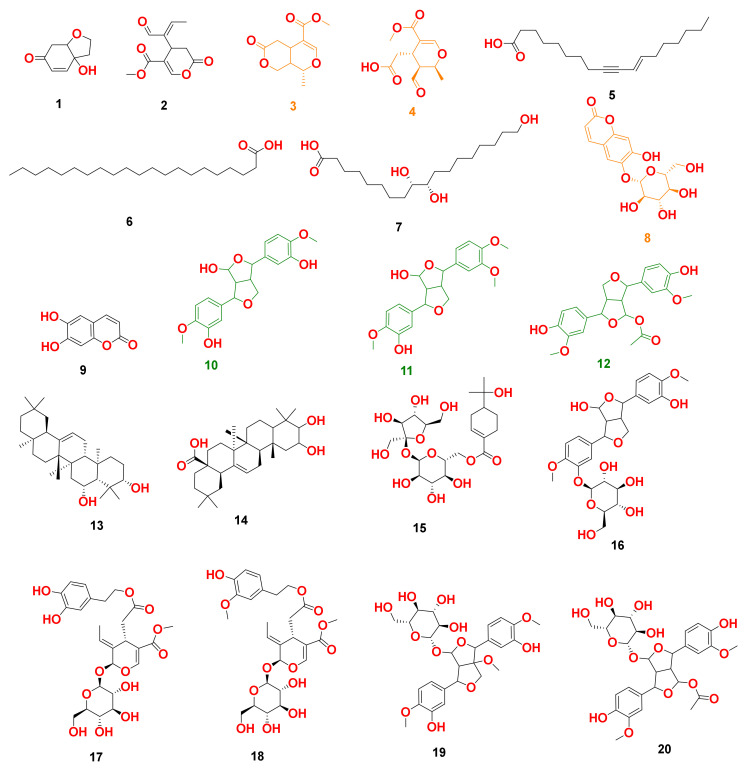
Dereplicated metabolites from LC-HRESIMS analysis of *Olea europaea.* Orange-colored structures were predicted to bind to GR’s active site, while the green-colored structures were predicted to bind to SOD’s active site.

By analyzing OLE, several hits were proposed (Table 1, Figure 1, Figure 2 and Figure 3). The molecular ion mass peaks at *m*/*z* 155.0708 and 223.06055 [M + H]^+^, [M − H]^+^, respectively, for the predicted molecular formulas C_8_H_10_O_3_ and C_11_H_12_O_5_ gave hits of megaritolactones, and *S*-(*E*)-elenolide, halleridone **1**, and elenolide **2**, respectively, that were formerly isolated from *O. europaea* [31,32].

The mass ion peaks at *m*/*z* 227.09206, 243.0869, 277.2167, 327.3263, and 331.24803 corresponding to the suggested molecular formulas C_11_H_14_O_5_, C_11_H_14_O_6_, C_18_H_30_O_2_, C_21_H_42_O_2_, and C_18_H_36_O_5_ [M + H]^+^, [M − H]^+^, respectively, fit a fatty acid, and secoiridoid derivative compounds olenoside A **3**, elenaic acid **4**, 11-octadecen-9-ynoic acid **5**, heneicosanoic acid **6**, and 9,10,18-trihydroxyoctadecanoic acid **7**, which was previously isolated from *O. europaea* [33,34,35,36,37]. In addition, the mass ion peaks at *m*/*z* 341.08660, 341.0873, 375.1444, 389.1600, and 417.15389 corresponding to the suggested molecular formulas C_15_H_16_O_9_, C_15_H_16_O_9_, C_20_H_22_O_7_, C_21_H_24_O_7_, and C_22_H_24_O_8_ [M + H]^+^ fit a benzopyrane and tetrahydrofuran lignan derivative compounds 6,7-dihydroxy-2H-1-benzopyran-2-one; 6-*O-β*-D-glucopyranoside **8**, 6,7-dihydroxy-2H-1-benzopyran-2-one; 6-*O-β*-D-glucopyranoside **9**, 7,9′:7′,9-diepoxy-8,8′-lignan-3,3′,4,4′,8-pentol; 3,3′-di-Me ether **10**, 7,9′:7′,9-diepoxy-8,8′-lignan-3,3′,4,4′,8-pentol; 3,3′,4′-tri-Me ether **11**, and 3,3′,4,4′,8-pentahydroxy-7,9′:7′,9-diepoxylignan; 3,3′-Di-Me ether, 8-Ac **12**, which was previously isolated from *O. europaea* [38,39,40].

Moreover, the molecular ion mass peaks at *m*/*z* 443.3889 and 473.36213 [M + H]^+^, for the predicted molecular formulas C_30_H_50_O_2_ and C_30_H_48_O_4_, gave hits of the triterpenes, 12-oleanene-3,28-diol **13**, and 2,3-dihydroxy-13(18)-oleanen-28-oic acid **14**, respectively, which were previously isolated from *O. europaea* [41].

The ion mass peaks at *m*/*z* 509.22142, 537.1972, 541.1921, and 555.2078 [M + H]^+^ for the predicted molecular formulas C_22_H_36_O_13_, C_26_H_32_O_12_, C_25_H_32_O_13_, and C_26_H_34_O_13_, respectively, gave hits of the 6-*O*-oleuropeoylsucrose **15**, which was isolated from *O. europaea* [4], the tetrahydrofuran lignan nucleus of 7,9′:7′,9-diepoxy-8,8′-lignan-3,3′,4,4′,8-pentol, 3,3′-Di-Me ether, and 4-*O-β*-D-glucopyranoside 1**6**, which was isolated from *O. europaea* [35], and secoiridoid derivative compounds oleuropein **17** and oleuropein; 4″-Me ether **18**, which were isolated from *O. europaea* [41,42]. Two main ion peaks with the *m*/*z* values of 567.2078 and 579.20783 [M + H]^+^ with molecular formulas C_27_H_34_O_13_ and C_28_H_34_O_13_, respectively, were detected and dereplicated as 7,9′:7′,9-diepoxy-8,8′-lignan-3,3′,4,4′,5,8-hexol, 3,3′,5-tri-Me ether, 8-*O-β*-D-glucopyranoside 1**9**, 3,3′,4,4′,8-pentahydroxy-7,9′:7′,9-diepoxylignan, 3,3′-di-Me ether, and 8-Ac, 4-*O-β*-D-glucopyranoside **20**, respectively, which were isolated earlier from *O. europaea* [4].

### 3.2. OLE-Containing Spanlastics

**Figure 4:**
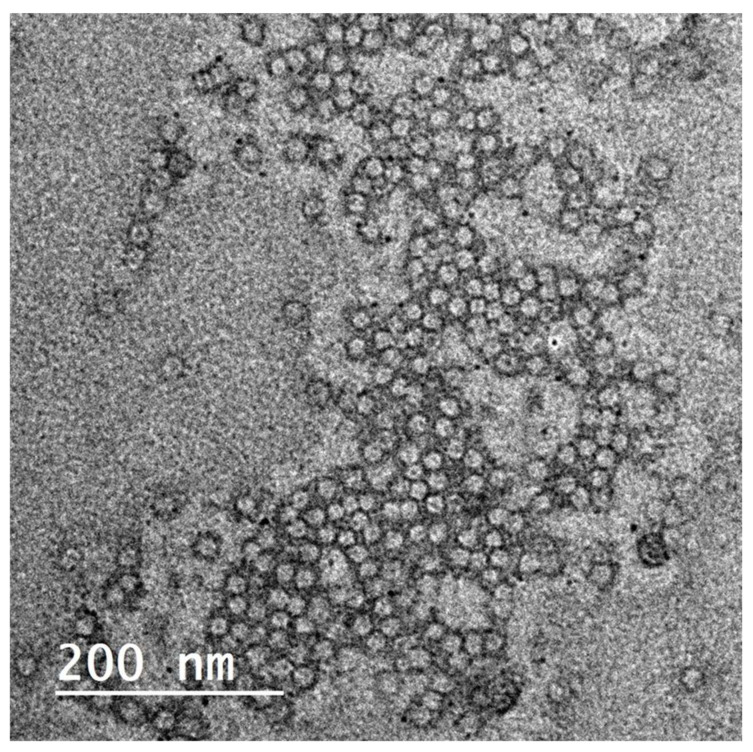
TEM image for *Olea europaea* ethanolic extract-containing spanlastics.

Elastic nanovesicles, so-called spanlastics of OLE, were successfully prepared. The TEM image shows the vesicular structure of the formed spanlastics (Figure 4). The vesicles-obtained particle size of 41 ± 2.4 nm and PDI 0.279. The Zeta potential of the prepared spanlastics was 13.6 ± 2.5. The small size and the narrow size distribution of the formulation were achieved by the adopted spraying method for preparation. Spraying of the organic phase onto the aqueous phase surface provided an enlarged surface area and improved wettability of the sprayed organic phase 13, 44. The formulated spanlastics entrapped 61.43 ± 2.03% of the loaded extract. Adding ethanol during formulation contributed to the enhancement of the drug partitioning and entrapment within the spanlastic vesicles. Moreover, the structure of the spanlastics imparted better inclusion of both the hydrophilic ingredients and the lipophilic ingredients of the extract within the core of the vesicle or the bilayered membrane, respectively.

**Figure 5:**
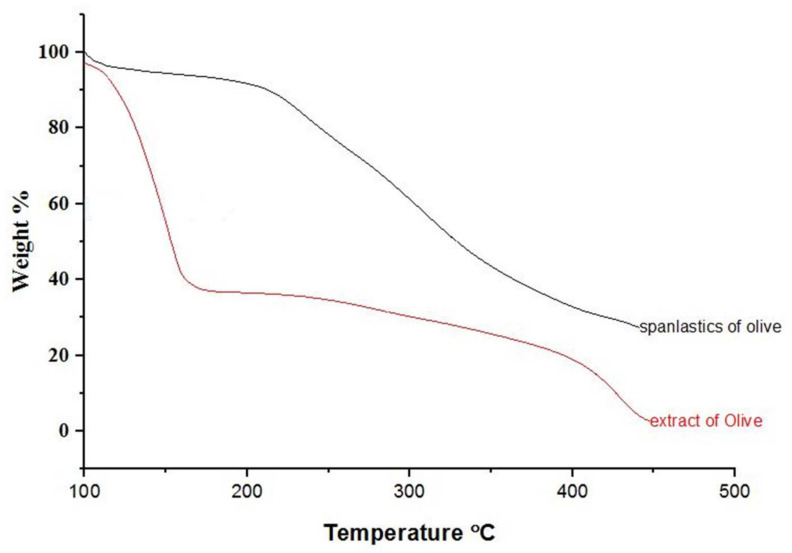
TGA spectra of olive extract and spanlastics of olive.

Physicochemical changes in compounds as a function of temperature were evaluated by TGA. TGA curves for OLE and OLE spanlastics are shown in Figure 5. During heating from 30 to 450 °C, about 62 and 13% weight loss was detected at a temperature of 126 °C for OLE and OLE spanlastics, respectively. Weight loss may be due to the phase transition caused by a change in the molecular structure. Results reveal the enhanced thermal balance of the entrapped cargo owing to spanlastic encapsulation.

**Figure 6:**
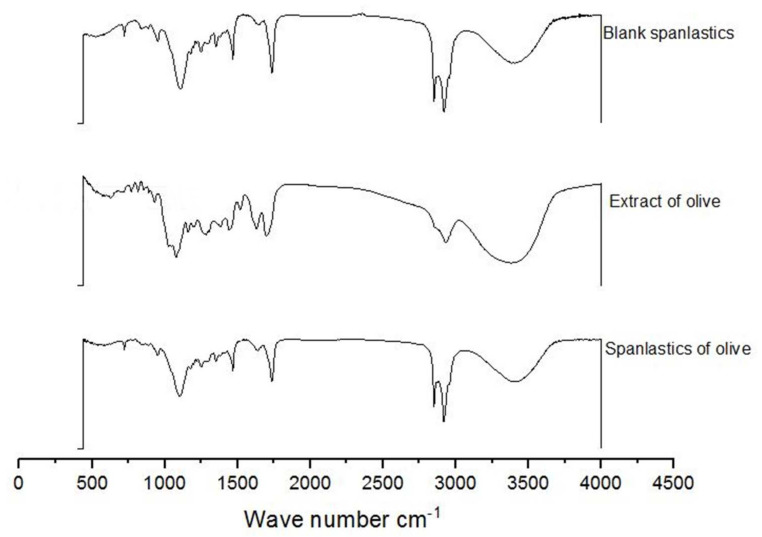
FTIR spectra of blank spanlastics, olive extract, and spanlastics of olive.

FTIR spectra of OLE, blank spanlastics, and OLE spanlastics were studied to understand the association between components of OLE and components of the spanlastic membrane (Span 60 and Tween 80) (Figure 6). The FTIR spectrum of OLE contains principal bands at 3391, 2942, 1705, 1499, and 1090, and that of Blank spanlastics contains bands at 2928, 2870, 1756, 1481, and 1105. The FTIR spectrum of olive spanlastics contains no extra bands to those involved in both free extract and blank spanlastics spectra, suggesting that the spanlastics encapsulation of OLE within the spanlastics did not set-up new connections.

### 3.3. Antiproliferative Activity of the OLE and OLE-Spanlastics

Many studies [43,44] support our findings in the context of the antiproliferative activity of the OLE. In this study, OLE was screened in vitro for its antiproliferative activity against hepatic, breast, and colorectal cancer cell lines (HepG-2, MCF-7, and Caco-2, respectively) and normal fibroblast cell line (WI-32). Results showed that OLE was able to moderately prohibit the growth of all tested cancer cell lines with IC_50_ equal to 9.2 ± 0.8, 7.1 ± 0.9, and 6.5 ± 0.7 µg/mL, respectively (Table 2).

**Figure 7:**
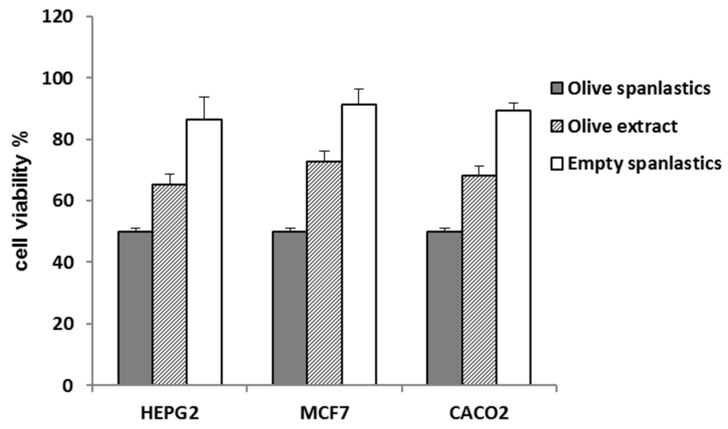
Cell viability of olive spanlastics and empty spanlastics against HepG-2. MCF-7, and Caco-2.

The spanlastic formulation improved the antiproliferative potential of the entrapped extract. IC_50_ values of the formulated extract were 3.6 ± 0.2, 2.3 ± 0.1, and 1.8 ± 0.1 µg/mL against HepG2, MCF-7, and Caco-2 (Table 2, Figure 7), respectively, *p* < 0.001.

That may be attributed to the concept of enhanced endocytosis of nanosized particles compared to larger ones [45,46]. Endocytosis of tiny particles needs less energy than that required for larger particles [47]. That means nanosized particles have a better chance for cellular uptake relative to larger counterparts [47]. Herein, cell viability was utilized as a tool to evaluate the extent of extract-containing spanlastic uptake compared to that of the crude extract (Table 2, Figure 7).

### 3.4. In Silico Study

Target Prediction and Docking Analysis: To propose a probable mode of action for OLE, all annotated compounds (Figure 3) were subject to a comprehensive inverse docking against almost all proteins introduced in the Protein Data Bank using the idTarget docking platform. This online-based molecular modeling software applies a unique virtual screening approach: divide-and-conquer docking. This approach adaptively builds small overlapping grids to improve the speed and accuracy of searching the space on protein surfaces, and thus, it can carry out an enormous number of precise docking experiments in a significantly reduced time [48]. In this docking platform, the query structure can be docked against most of the PDB proteins. Thereafter, the retrieved binding affinity scores of the docked proteins were arranged from the highest negative value to the lowest one.

We set a binding affinity score of −7 kcal/mol as a cut-off value to select the best scoring protein hits. All targets with scores lower than −7 kcal/mol were then carefully investigated to select cancer-related human proteins. Glutathione reductase (GR) showed very interesting binding scores with compounds **3**, **4**, and **8** (−11.35, −10.63, −9.87 kcal/mol, respectively), while superoxide dismutase (SOD) was a very good binder to compounds **10**, **11**, and **12** (−10.22, −9.56, and −9.11 kcal/mol, respectively).

**Figure 8:**
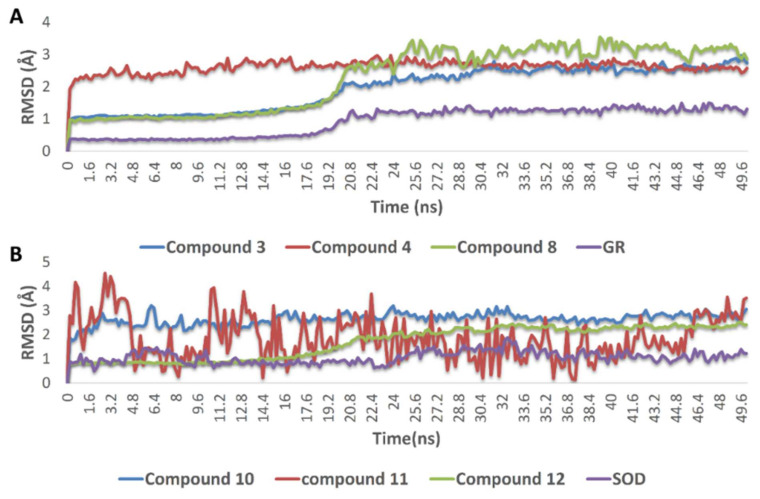
(**A**) RMSDs of compounds **3**, **4**, and **8** inside the active site of GR, and compounds **10, 11**, and (**B**) **12** inside the active site of SOD over 50 ns of MDS.

The docking poses of the previously mentioned compounds with GR and SOD were then subjected to 50 ns MDS experiments to study their stability inside the active site of each enzyme and to calculate their binding free energies (ΔG). As shown in Figure 8, compounds **3, 4, 8, 10, 11,** and **12** achieved very good binding stabilities inside the active site of both GR and SOD over the simulation time with very low deviations from the starting binding orientations (i.e., docking poses) (RMSD ~ 2.5 Å). Additionally, they achieved very promising ΔG values (−9.45, −8.36, −9.14, −9.76, −10.43, and −8.71 kcal/mol, respectively) as these indicate that they are very good binders to both enzymes. The last snapshot of each MDS was then extracted to investigate the attached mode of each compound inside the effective site of both enzymes.

**Figure 9:**
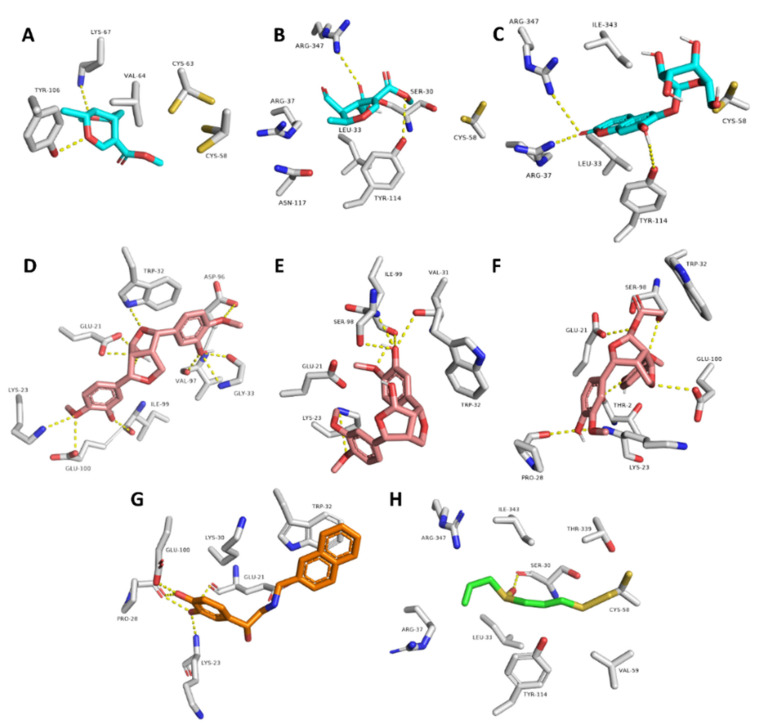
Binding mode of compounds **3**, **4**, and **8** inside the active site of GR ((**A**–**C**), respectively; brick-red-colored), Scheme **10**, **11**, and **12** ((**D**–**F**), respectively; brick red-colored structures) inside the active site of SOD, in addition to the binding mode of SOD and GR co-crystalized ligands ((**G**) and (**H**), respectively).

Moreover, compounds **3**, **4**, and **8** showed multiple stable H-bonds, particularly with ARG-37, LYS-67, TYR-106, TYR-114, and ARG-347 inside the active site of GR, while compounds **10**, **11**, and **12** exhibited a higher number of H-bonds inside the active site of SOD, where GLU-21, LYS-23, TRP-32, and SER-98 were the common amino acids involved in these interactions (Figure 9).

These two oxidative stress-related enzymes were selected as probable targets for OLE based upon many previous reports that have shown a direct link between the inhibition of these enzymes and the elevated cellular oxidative stress that can eventually lead to cancer cell death. This mechanism of anticancer activity is also very selective because cancer cells are typically highly susceptible to raised oxidative stress in contrast to healthy cells [49,50,51,52,53].

### 3.5. Pro-Oxidant Activity of OLE and OLE Spanlastic

**Figure 10:**
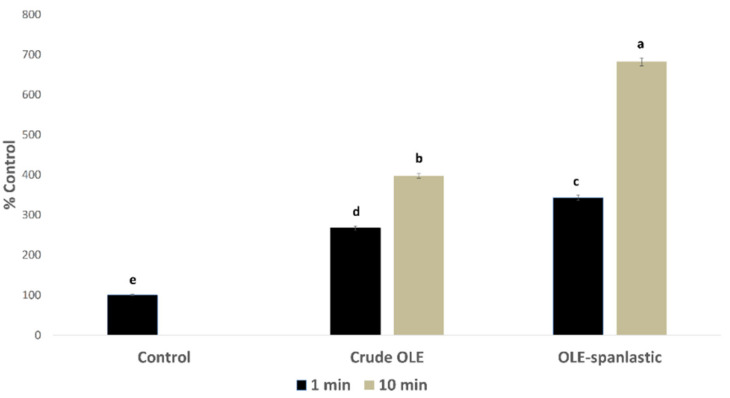
Free radical production induced by OLE and OLE-spanlastics in Caco-2 cells. The test cells were treated (*n* = 3) with 7 µg/mL from each test material. Intracellular free radical levels were determined after 1 min and 10 min by using the DCFDA fluorescent method, and the results were expressed as the % produced fluorescence in respect of that of the control (% Control). Bar graphs represent the mean of 3 independent experiments, where the small letters (a, b, c, d, and e) indicate significance between groups (*p* < 0.001).

In silico investigation of the OLE revealed its potential to exhibit an intracellular pro-oxidant activity. In this study, OLE and its derived spanlastics were screened in vitro for measuring the percentage of free radicals’ production inside Caco-2 cells using DCFDA assay. Results showed a significant rise in the free radical production in Caco-2 cells upon treatment with 7 µg/mL of both OLE and OLE-spanlastics, with %DCF 267 ± 5.15 and 343 ± 4.95, respectively, after 1 min of treatment, and %DCF 398 ± 5.91 and 682 ± 7.17, respectively, after 10 min, compared to the untreated cells (i.e., normal control) (Figure 10).

Furthermore, we tested OLE for its inhibitory potential toward the enzymatic activity of both GR and SOD to test if these two enzymes that protect cells from elevated free radicals were targeted by OLE chemical components.

Glutathione reductase (GR), or the glutathione-disulfide reductase (GSR) enzyme, catalyzes the reduction of glutathione disulfide (GSSG) to the sulfhydryl form glutathione (GSH), which is a critical molecule in resisting oxidative stress and maintaining the reducing environment of the cell^53^. Glutathione anions act on a water-soluble formazan dye to produce a color that can be distinguished by the rise in absorbance at 450 nm. The higher the activity of GR in the sample, the lower the amount of formazan dye that is formed. In this study, OLE and its derived spanlastics were screened in vitro for their inhibitory potential toward the enzymatic activity of GR colorimetrically using the manufacturer’s protocol (abcam, Cat.No: ab8346). The results showed that OLE and its derived spanlastics were able to inhibit the activity of the GR enzyme significantly with IC_50_ values of 1.18 ± 0.12 µg/mL and 0.54 ± 0.11, respectively.

In addition, superoxide dismutase (SOD) is one of the most remarkable antioxidant enzymes. It catalyzes the dismutation of the superoxide anion produced by xanthin oxidase into hydrogen peroxide and molecular oxygen^52^. As with GR, superoxide anions act on a water-soluble formazan dye to produce a color that can be distinguished by the rise in absorbance at 450 nm. The higher the activity of SOD in the sample, the lower the amount of formazan dye that is formed. In this study, OLE and its derived spanlastics were screened in vitro for their inhibitory potential toward the enzymatic activity of SOD colorimetrically using the manufacturer’s protocol (abcam, Cat.No: ab65354). The results showed that OLE and its derived spanlastics were able to inhibit the activity of the SOD enzyme significantly with IC_50_ values of 2.33 ± 0.19 µg/mL and 0.73 ± 0.23, respectively.

## 4. Conclusions

OLE showed mild in vitro antiproliferative activity against HepG-2, MCF-7, and Caco-2, which were enhanced through the entrapment of OLE within spanlastic vesicles. In silico and modeling investigation annotated compounds in OLE to interact with a number of oxidative stress-protecting enzymes (i.e., GR and SOD). In vitro testing supported these findings and uncovered the pro-oxidant capacity of OLE and its derived spanlastics to be able to induce intracellular free radicals in Caco-2 cells and significantly inhibit the enzymatic activity of both GR and SOD. Our findings in the present study highlight the potential of OLE and its nanoformulation (i.e., spanlastics) as promising pro-oxidant anticancer agents.

## Figures and Tables

**Table 1:** Dereplicated metabolites from LC-HRESIMS analysis of *Olea europaea* L. cv. Arbosana.

Nu.	Metabolite Name	Original Source	MF	RT (min.)	*m/z*
**1**	Halleridone	*Olea europaea*	C_8_H_10_O_3_	3.78010	155.0708
**2**	Elenolide	*Olea europaea*	C_11_H_12_O_5_	2.20205	223.06055
**3**	Olenoside A	*Olea europaea*	C_11_H_14_O_5_	2.27261	227.09206
**4**	Elenaic acid	*Olea europaea*	C_11_H_14_O_6_	4.18760	243.0869
**5**	11-Octadecen-9-ynoic acid	*Olea europaea*	C_18_H_30_O_2_	6.73612	277.2167
**6**	Heneicosanoic acid	*Olea europaea*	C_21_H_42_O_2_	6.01750	327.3263
**7**	9,10,18-Trihydroxyoctadecanoic acid	*Olea europaea*	C_18_H_36_O_5_	4.09611	331.24803
**8**	6,7-Dihydroxy-2H-1-benzopyran-2-one; 6-*O-β*-D-Glucopyranoside	*Olea europaea*	C_15_H_16_O_9_	2.37151	341.08660
**9**	6,7-Dihydroxy-2H-1-benzopyran-2-one	*Olea europaea*	C_9_H_6_O_4_	3.78010	178.0264
**10**	7,9′:7′,9-Diepoxy-8,8′-lignan-3,3′,4,4′,8-pentol, 3,3′-Di-Me ether	*Olea europaea*	C_20_H_22_O_7_	2.01432	375.1444
**11**	7,9′:7′,9-Diepoxy-8,8′-lignan-3,3′,4,4′,8-pentol, 3,3′,4′-Tri-Me ether	*Olea europaea*	C_21_H_24_O_7_	2.01740	389.1600
**12**	3,3′,4,4′,8-Pentahydroxy-7,9′:7′,9-diepoxylignan, 3,3′-Di-Me ether, 8-Ac	*Olea europaea*	C_22_H_24_O_8_	3.23649	417.15389
**13**	12-Oleanene-3,28-diol	*Olea europaea*	C_30_H_50_O_2_	5.90870	443.3889
**14**	2,3-Dihydroxy-13(18)-oleanen-28-oic acid	*Olea europaea*	C_30_H_48_O_4_	5.96396	473.36213
**15**	6-*O*-Oleuropeoylsucrose	*Olea europaea*	C_22_H_36_O_13_	2.49208	509.22142
**16**	7,9′:7′,9-Diepoxy-8,8′-lignan-3,3′,4,4′,8-pentol, 3,3′-Di-Me ether, 4-*O-β*-D-glucopyranoside	*Olea europaea*	C_26_H_32_O_12_	2.40519	537.1972
**17**	Oleuropein	*Olea europaea*	C_25_H_32_O_13_	3.02400	541.1921
**18**	Oleuropein; 4″;-Me ether	*Olea europaea*	C_26_H_34_O_13_	3.02300	555.2078
**19**	7,9′:7′,9-Diepoxy-8,8′-lignan-3,3′,4,4′,5,8-hexol, 3,3′,5-Tri-Me ether, 8-*O-β*-D-glucopyranoside	*Olea europaea*	C_27_H_34_O_13_	2.53020	567.2078
**20**	3,3′,4,4′,8-Pentahydroxy-7,9′:7′,9-diepoxylignan, 3,3′-Di-Me ether, 8-Ac, 4-*O-β*-D-glucopyranoside	*Olea europaea*	C_28_H_34_O_13_	2.75095	579.20783

MF: molecular formula; RT (min.): retention time per mint, *m/z*: mass to charge.

**Table 2:** In vitro antiproliferative activity of *Olea europaea* ethanolic crude extract shown as IC_50_ ± (SD) µM.

IC_50_ (µM)				
Code	HepG-2	MCF-7	Caco-2	WI-38
**OLE**	9.2 ± 0.8	7.1 ± 0.9	6.5 ± 0.7	>50
**OLE-spanlastic**	3.6 ± 0.2 ***	2.3 ± 0.1 ***	1.8 ± 0.1 ***	44 ± 0.2
**Doxorubicin**	4.2 ± 0.3	3.8 ± 0.2	3.4 ± 0.1	27.32 ± 0.2

The IC_50_ value of OLE crude extract and its derived spanlastics (OLE-spanlastic) against three human cancer cell lines, along with normal human fibroblasts (i.e., WI-38), described as the concentration (µM) that led to in vitro inhibition of cell growth in a percentage of 50%. Data were given as mean ± SEM (*n* = 3), *** *p* < 0.001 compared to crude unprocessed extract.

## Data Availability

Data are contained within the article.
